# Comprehensive analysis of VOZ proteins in sweet potato and related species reveals their evolutionary dynamics and responses to abiotic stresses

**DOI:** 10.3389/fpls.2026.1775128

**Published:** 2026-03-13

**Authors:** Zhidan Zuo, Yeshun Sheng, Chenglin Jia, Huihui Ma, Yuxin Wang

**Affiliations:** 1 College of Life Sciences, Zaozhuang University, Zaozhuang, China; 2 Key Laboratory of Sweet Potato Biology and Biotechnology of Ministry of Agriculture and Rural Affairs, College of Agronomy and Biotechnology, China Agricultural University, Beijing, China

**Keywords:** evolutionary and phylogenetic analysis, expression patterns, *Ipomoea* species, protein interaction network, sweet potato, VOZs

## Abstract

VOZ (Vascular Plant One-Zinc Finger) transcription factors represent a plant-specific family of regulatory proteins that are pivotal in controlling plant growth, developmental processes, and adaptive responses to biotic and abiotic stresses. Although *VOZ* genes have been reported in multiple plant species, their genomic organization, evolutionary history, and functional dynamics in sweet potato remain largely unexplored. In this study, a comprehensive genome-wide investigation of *VOZ* family members was conducted across six *Ipomoea* species, namely *I. aquatica* (Iaq), *I. cairica* (Ica), *I. nil* (Inil), *I. triloba* (Itb), *I. trifida* (Itf), and *Ipomoea batatas* (Ib). In terms of their phylogenetic relationships, protein properties, gene architecture, conserved motifs, promoter *cis*-elements, chromosomal localization, and collinearity patterns, 14 *VOZ* genes were identified and systematically analyzed. The findings reveal a contraction in *VOZ* copy number, accompanied by structural and functional divergence during the evolutionary trajectory of these *Ipomoea* species. Furthermore, transcriptional profiling and protein-protein interaction network characterization in sweet potato indicate that *VOZs* from *I. trifida* (ItfVOZs) and *I. batatas* (*IbVOZs*) are implicated in developmental regulation, hormone-mediated signaling pathways, and stress adaptation. Collectively, this study provides a comprehensive genomic framework for the *VOZ* gene family across six *Ipomoea* species and provides a solid foundation for elucidating their functional roles in sweet potato.

## 1 Introduction

Transcription factors (TFs) are the major regulatory proteins that play vital roles in plant life processes. They regulate gene expression by binding to the *cis-*elements of promoters in their target gene or through protein-protein interactions to perform their functions (Jiang and Yu, 2015; Fang and Chen, 2024). With continued advances in high-throughput sequencing technologies, genome-wide comparative analysis has become an important tool in plant genomics research (Ji et al., 2006; Wang et al., 2023). An expanding body of studies has applied this strategy to systematically investigate TF families across diverse plant species, thereby providing a robust theoretical framework for functional annotation and evolutionary inference. Comparative analyses of MYB TFs have enhanced understanding of their evolutionary diversification and functional roles in rice and *Arabidopsis* (Katiyar et al., 2012). In *Salvia miltiorrhiza*, 110 *R2R3-MYBs* were identified and characterized, revealing their potential regulatory involvement in the biosynthesis of bioactive secondary metabolites (Li and Lu, 2014). Genome-wide comparative studies in *Arabidopsis* and rice have demonstrated that the WRKY, MADS-box, and MYB TF families are critically involved in plant growth, development, and environmental stress response (Abdullah-Zawawi et al., 2021).

VOZ TFs are key vital regulators of plant growth, development and stress responses. It was first identified in *Arabidopsis*, which contained two conserved regions, namely Domain-A and Domain-B (Mitsuda et al., 2004; Ganie et al., 2020). *VOZs* exert critical functions in flowering regulation via several mechanisms. For example, AtVOZ2 binds to the GCGTNx7ACGC sequence within the *cis*-regulatory region of the pollen-specific *AVP1* gene, thereby contributing to pollen development (Mitsuda et al., 2003, Mitsuda et al., 2004). Both AtVOZ1 and AtVOZ2 associate with phytochrome B and CONSTANS (CO) to coordinate flowering timing (Yasui et al., 2012; Kumar et al., 2018). Furthermore, AtVOZs modulate the transcription of FLOWERING LOCUS C (FLC) and *MOS3/SAR3*, thereby regulating floral initiation (Celesnik et al., 2013; Yasui and Kohchi, 2014). The PHYB-FOF2-VOZ2 regulatory module refines flowering progression by controlling *FLC* expression (Qu et al., 2024). In tomato, the SlOST1–SlVOZ1 module regulates flowering under drought conditions, with SlVOZ1 directly binding to the promoter of the principal flowering integrator gene *SINGLE FLOWER TRUSS* (Chong et al., 2022). In Satsuma mandarin, CuVOZ2 initiates precocious flowering by coordinating the interplay between vegetative and reproductive development via interactions with FT proteins (Hasan et al., 2023). *VOZs* are also instrumental in biotic and abiotic stress responses. *AtVOZ1* and *AtVOZ2* contribute to plant immunity in *Arabidopsis* (Schwarzenbacher et al., 2020). In rice, *OsVOZ2* mediates resistance against bacterial blight, whereas *OsVOZ1* and *OsVOZ2* function as adaptors linking ubiquitin ligases to regulate blast resistance (Cheong et al., 2013; Wang et al., 2021). In *Arabidopsis, AtVOZ1* suppresses *DREB2C* transcription, thereby negatively regulating heat stress responses, whereas *AtVOZ2* overexpression improves biotic stress resilience but compromises abiotic stress tolerance (Nakai et al., 2013; Song et al., 2018). *VOZ* TFs further control arsenic tolerance and distribution in rice and *Arabidopsis* (Wen et al., 2023). In soybean, *GMVOZ1G* enhances resilience to drought and salinity (Li et al., 2020).

*Ipomoea* is one of the largest and most complex genus in the world. Hexaploid sweet potato (*Ipomoea batatas* (L.) Lam., 2n = B1B1B2B2B2B2 = 6x = 90) is a universally significant, high-producing, and nutrient-rich root crop (Munoz-Rodriguez et al., 2019; Kwak, 2019). It has diverse applications in human nutrition, animal feed, starch production, and industrial use, providing considerable potential to enhance food and nutritional security (Liu, 2017; Zhang et al., 2022; Jiang et al., 2023). Despite its agronomic importance, genetic breeding of sweet potato is hindered by several challenges, including its large genome size, high ploidy level, heterozygosity, complex chromosome architecture, and self-incompatibility (Arumuganathan and Earle, 1991; Yan et al., 2022). Recent advances in genome sequencing have facilitated the identification and functional characterization of major genes in sweet potato. High-quality genome assemblies of *I. trifida, I. triloba*, and the hexaploid variety *I. batatas* “Taizhong6, ” serve as comprehensive genomic references for functional and comparative studies (Hirakawa et al., 2015; Yang et al., 2017; Wu et al., 2018). Genome-wide comparative analyses have become a fundamental strategy for characterizing gene families (Wei et al., 2002). Numerous key gene families–such as glutathione S-transferases, valine-glutamine motif-containing genes, DMP genes, MTL genes, and xyloglucan endotransglucosylase/hydrolase genes–have been extensively examined and functionally annotated in sweet potato (Ding et al., 2017; Si et al., 2023a; Zhang et al., 2023; Pan et al., 2024). However, the VOZ TF family in sweet potato has not yet been uncharacterized.

The *VOZ* gene family was analyzed across six *Ipomoea* species. Fourteen *VOZ* genes were identified—three in *I. aquatica*, three in *I. cairica*, two in *I. nil*, two in *I. triloba*, two in *I. trifida*, and two in *I. batatas*—representing both diploid and hexaploid members of the genus. Genes were grouped into three distinct subfamilies. The evolutionary and phylogenetic relationships, protein properties, gene structures, promoter *cis*-elements, conserved motifs, chromosomal localizations, and genomic distribution were characterized using comprehensive analyses. In addition, their expression profiles and protein–protein interaction networks were investigated. These findings provide a robust theoretical framework for future functional characterization of *VOZ* genes in sweet potato.

## 2 Materials and methods

### 2.1 Identification of *VOZs* in the six *Ipomoea* species

Genome sequences of *I. aquatic*a (*Iaq*), *I. cairica* (*Ica*), and *I. nil* (*Inil*) were retrieved from the National Genomics Data Center (NGDC) (https://ngdc.cncb.ac.cn/gwh/Assembly/986/show), the Zenodo repository (https://zenodo.org/records/6792002#.Y90Mb3ZBy4Q), and the Shigen database (http://viewer.shigen.info/asagao/index.php), respectively. Genome assemblies of *I. triloba* (*Itb*)*, I. trifida* (*Itf*), and *I. batatas* (*Ib*) were obtained from the *Ipomoea* Genome Hub (https://www.sweetpotao.com/). *VOZ* genes were identified using the BLAST database. Amino acid sequences of *VOZs* from *Arabidopsis thaliana* were used as queries in the BLASTP analyses, with an E-value threshold of ≤ 1×10^−5^. All candidate VOZ proteins were subsequently validated using InterProScan (https://www.ebi.ac.uk/interpro/).

### 2.2 Characterization of the *VOZs*

The physicochemical properties of VOZ proteins were computed using ExPASy ProtParam (https://web.expasy.org/protparam/). Subcellular localization was predicted using DeepLoc 2.1 (https://services.healthtech.dtu.dk/services/DeepLoc-2.1/).

### 2.3 Prediction of protein secondary and three-dimensional structures

The secondary structures of VOZ proteins were predicted using NetSurfP-3.0 (https://services.healthtech.dtu.dk/services/NetSurfP-3.0/), while three-dimensional (3-D) structures were modeled using AlphaFold3 (Høie et al., 2022; Abramson et al., 2024). The conserved domain architectures were visualized using PyMOL v3.13 (Mooers, 2020).

### 2.4 Evolutionary and phylogenetic analyses

The phylogenetic relationships of *VOZs* across the six were inferred using MAFFT (v7.307) with *Ipomoea* default parameters (Katoh et al., 2005). The resulting multiple sequence alignment was trimmed using TrimAl (v1.4) with default settings (Capella-Gutiérrez et al., 2009). A maximum-likelihood phylogenetic tree was constructed using IQ-TREE 2, incorporating 1, 000 bootstrap replicates and automatic selection of the best-fit substitution model (Minh et al., 2020). The Interactive Tree of Life (iTOL) platform (https://itol.embl.de/index.shtml) was used for tree visualization.

### 2.5 Gene structure and conserved analysis of *VOZ*s

Conserved motifs in VOZ proteins were detected using the Multiple Expectation Maximization for Motif Elicitation (MEME) suite with default settings (https://meme-suite.org/meme/tools/meme). Ten distinct VOZ motifs were identified. Gene structures were visualized using the Gene Structure Display Server (GSDS) (http://gsds.gao-lab.org/).

### 2.6 Collinearity analysis and gene duplication classification

The BLASTP results were processed using MCScanX (v1.0.0) with the default settings to identify collinearity blocks across the genomes (Wang et al., 2012). Collinear gene pairs were retrieved, and a genomic collinearity map was constructed using CIRCOS (Krzywinski et al., 2009). Gene duplication events were classified using the duplicate_gene_classifier script incorporated in MCScanX.

### 2.7 *Cis*-elements analysis of the *VOZs* promoter

A 2, 000-bp sequence upstream of the transcription start site of each *VOZ* gene was extracted using TBtools v2.400 (Chen et al., 2023). The PlantCARE database (http://bioinformatics.psb.ugent.be/webtools/plantcare/html/) was used to predict *cis*-regulatory elements in these promoter regions. The identified *cis*-elements were visualized using TBtools and the Seaborn Python package.

### 2.8 Tissue-specific expression patterns of *VOZs*

To examine the expression profiles of *VOZs* in sweet potato, publicly available RNA sequencing (RNA-Seq) datasets from BioProject accessions PRJCA000640 and PRJNA511028 in the NGDC were retrieved. Adapter sequences and low-quality reads were filtered using the Fastp (v1.1.0) default settings (Chen et al., 2018). The cleaned reads were subsequently aligned to the sweet potato reference genome (obtained from the *Ipomoea* Genome Hub) using STAR (v2.7.11b) with the default settings (Dobin et al., 2013). Read counts for exons were measured using FeatureCounts (v2.0.8) with the paired-end read counting option (-p -countReadPairs) (Liao et al., 2014). Transcript levels were represented as transcripts per kilobase of the exon model per million mapped reads (TPM) and were computed using a custom Python script. *VOZ* expression profiles across numerous tissues were standardized as log_2_ (TPM + 1), and a heatmap was visualized using the Python Seaborn package.

### 2.9 Prediction of VOZ protein interaction networks

Genome-wide protein–protein interactions of VOZ proteins were predicted using the STRING and AlphaFold 3 databases (https://cn.string-db.org/) (von Mering et al., 2003).

### 2.10 Subcellular localization

The IbVOZ1 coding sequence was cloned into the pCAMBIA1300-GFP vector to generate a pCAMBIA1300-IbVOZ1-GFP fusion construct. Subcellular localization markers fused to mCherry—specifically the nuclear marker NLS-mCherry and the plasma membrane marker PIP2A-mCherry—were co-introduced as references. Recombinant constructs were delivered into protoplasts and incubated for approximately 16 h. Fluorescence of GFP (488 nm) and mCherry (546 nm) was visualized using a confocal fluorescence microscope (Zeiss LSM900, Jena, Germany).

### 2.11 Transactivation activity assay

The IbVOZ1 coding sequence was inserted into the pBD-GAL4 vector to function as an effector. The firefly luciferase (LUC) gene was used as the reporter, and the renilla luciferase (REN) gene served as an internal control. These plasmids were simultaneously introduced into protoplasts via a Polyethylene glycol (PEG)-mediated delivery. Following the protocol described by Wang et al. (2025), LUC and REN activities were quantified.

### 2.12 Reverse transcription-quantitative real-time PCR analysis

To further profile the expression dynamics of *IbVOZ1*, 4-week-old *in vitro*-cultured sweet potato were subjected to 20% PEG6000 or 100 μM abscisic acid (ABA) for 0, 1, 3, 6, 12, and 24 h (Xue et al., 2022). Tissue-specific expression of *IbVOZ1* was additionally evaluated in leaves, roots, and stems of 4-week-old *in vitro*-grown plantlets, as well as in leaves, stems, fibrous roots, pencil roots, and storage roots of 3-month-old field-grown plants. Transcript abundance was assessed via RT-qPCR using SYBR Green Master Mix, with *IbACTIN* as the internal reference gene. The primer sequences are provided in **Supplementary Table 2**.

### 2.13 Statistical analysis

Data are given as mean ± SD and compared for significance using the two-tailed Student’ s t-test at *P* < 0.05 (*) and *P* < 0.01 (**) or ANOVA followed by posthoc Tukey’ s test at *P* < 0.05 (lowercase letters).

## 3 Results

### 3.1 Identification of the *VOZs* in the six *Ipomoea* species

**Figure 1:**
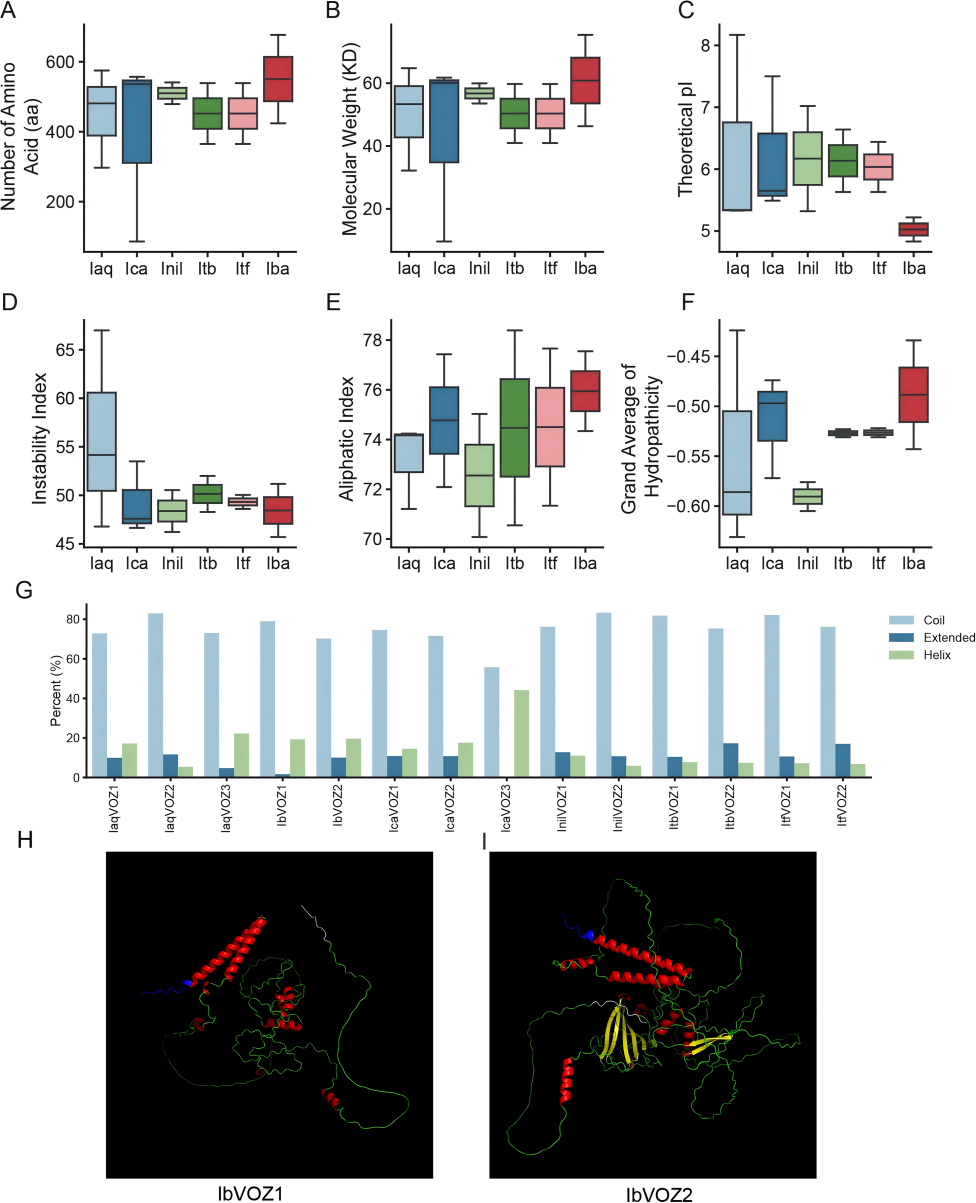
Analysis of the physicochemical properties of VOZ proteins in *I. aquatica*, *I. cairica*, *I. nil*, *I. triloba*, *I. trifida* and *I. batatas.***(A)** Number of amino acids of VOZ proteins. **(B)** Molecular weight of VOZ proteins. **(C)** Theoretical isoelectric point (pI) of VOZ proteins. **(D)** Instability index of VOZ proteins. **(E)** Aliphatic index of VOZ proteins. **(F)** Grand average of hydropathicity (GRAVY) of VOZ proteins. **(G)** Predicted secondary structures of VOZ proteins. **(H, I)** Predicted three-dimensional (3-D) structures of IbVOZ1 and IbVOZ2, respectively. *Ipomoea aquatica*, *Iaq*; *Ipomoea cairica*, *Ica*; *Ipomoea nil*, *Inil*; *Ipomoea triloba*, *Itb*; *Ipomoea trifida*, *Itf*; *Ipomoea batatas*, *Iba*. The ten amino acids at the starting position of the protein were labeled blue, and the ten amino acids at the ending position were labeled white.

To comprehensively identify *VOZ* genes in six *Ipomoea* species, homology-based BLAST was conducted. In total, 14 VOZ genes were detected across *I. aquatica* (*IaqVOZs*)*, I. cairica* (*IcaVOZs*)*, I. nil* (*InilVOZs*)*, I. triloba* (*ItbVOZs*)*, I. trifida* (*ItfVOZs*), and *I. batatas* (*IbVOZs*) (**Supplementary Table 1**). InterProScan verification demonstrated that all candidate proteins contained the conserved VOZ domain, confirming their annotation as VOZ family members. The coding sequence (CDS) lengths of the identified *VOZ* genes ranged from 261 to 2034 bp, encoding proteins ranging from 86 aa (IcaVOZ3) to 677 aa (IbVOZ2). Correspondingly, predicted molecular weights varied between 9.62194 kDa (IcaVOZ3) and 75.36887 kDa (IbVOZ2), while theoretical isoelectric points (*pI*) ranged from 4.83 (IbVOZ2) to 8.17 (IaqVOZ3) (**Figures 1A–C**). The instability index of VOZ proteins extends from 45.7 (IbVOZ1) to 67.0 (IaqVOZ3), indicating that most members are potentially unstable. The aliphatic index ranges from 70.08 (InilVOZ1) to 78.39 (ItbVOZ1), whereas the grand average of hydropathicity (GRAVY) is consistently negative, ranging from –0.631 (IaqVOZ2) to –0.424 (IaqVOZ3) (**Figures 1D–F**). Subcellular localization predictions indicated that most VOZ proteins, including IaqVOZ1, IaqVOZ2, IcaVOZ1, IcaVOZ2, InilVOZ1, ItbVOZ2, ItfVOZ2, and IbVOZ2, are nuclear-localized. Several proteins displayed multiple localization patterns, including triple localization in the cytoplasm, nucleus, and plastids (InilVOZ2, ItbVOZ1, and ItfVOZ1), dual localization in the cytoplasm and nucleus (IbVOZ1 and IcaVOZ3), and extracellular localization (IaqVOZ3) (**Supplementary Table 1**). Secondary structure prediction indicated that random coils predominate in all VOZ proteins, accounting for 55.81% (IcaVOZ3) to 83.36% (InilVOZ2) of the total structure (**Figure 1G**). The proportion of helix content ranges from 5.41% (IaqVOZ2) to 44.19% (IcaVOZ3), whereas β-strands constitute between 1.65% (IbVOZ1) and 17.26% (ItbVOZ2) (**Figure 1G**). Three-dimensional structural modeling revealed that VOZ proteins in sweet potato display high structural conservation at both the N- and C-terminal regions, while noticeable divergence occurs within the central regions. This structural variability may contribute to functional diversification and regulatory specificity among VOZ family members (**Figures 1H, I**).

### 3.2 Evolutionary and phylogenetic relationship of the *VOZs* in the six *Ipomoea* species

**Figure 2:**
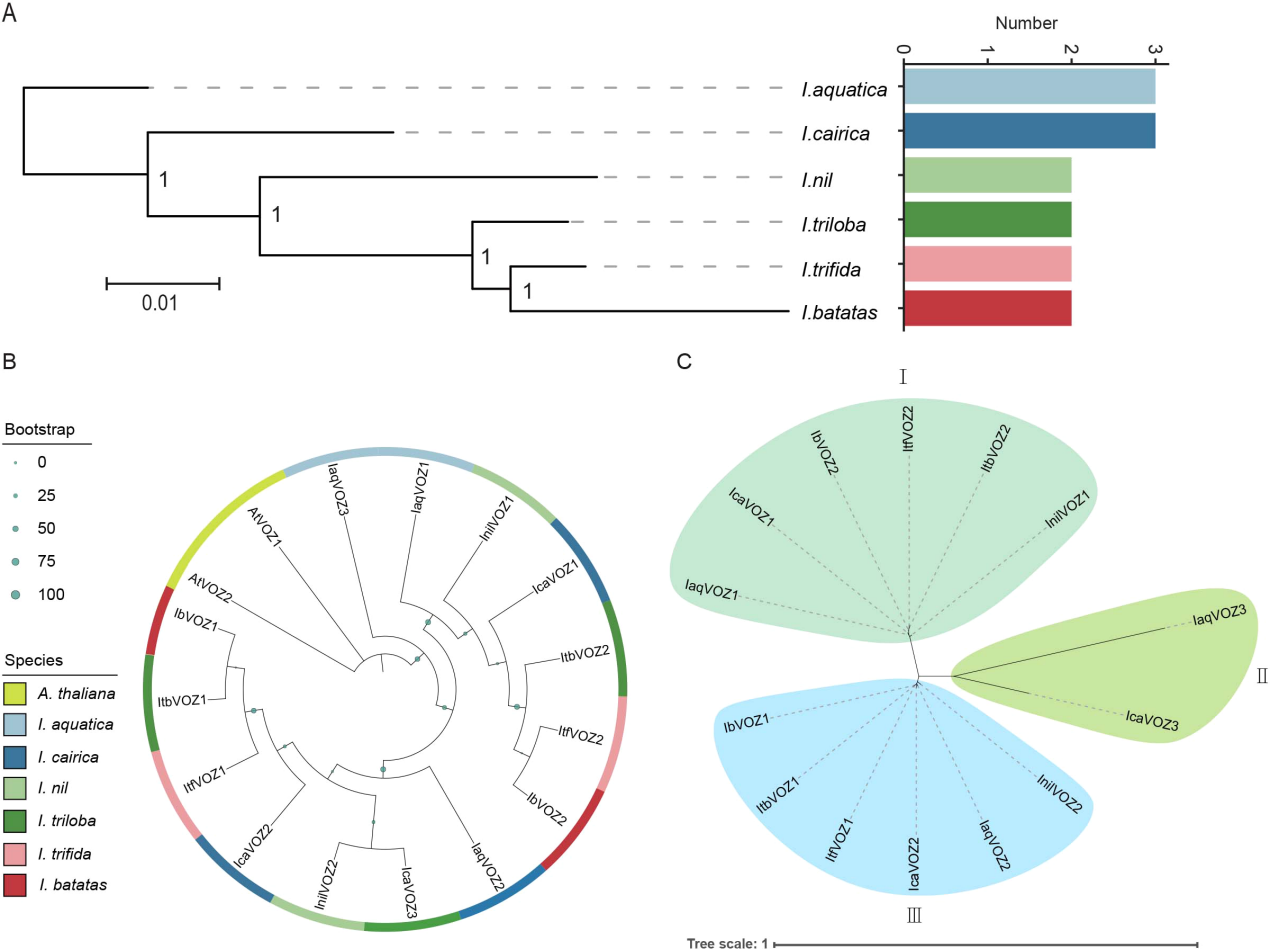
Evolutionary and phylogenetic analysis of the VOZs. **(A)** Evolutionary and phylogenetic analysis of the VOZs in *I. aquatica*, *I. cairica*, *I. nil*, *I. triloba*, *I. trifida* and *I. batatas*. The phylogenetic tree of VOZs in the six *Ipomoea* species is shown on the left and the number of members of different species is shown on the right. Values at the nodes indicate bootstrap support (1 = 100%). **(B)** The phylogenetic tree of VOZs in *Arabidopsis thaliana* (*A. thaliana*) and the six *Ipomoea* species. Different species were marked with different colors in the outer circles (AtVOZs, IaqVOZs, IcaVOZs, InilVOZs, ItbVOZs, ItfVOZs and IbVOZs were showed in yellow, light blue, dark blue, light green, dark green, pink and red respectively). **(C)** 14 VOZs were divided into three groups (groups I, II, and III filled with green, orange and blue, respectively).

Phylogenetic trees were constructed to elucidate the evolutionary history and phylogenetic relationships of *VOZ* genes across six *Ipomoea* species. *I. aquatica* and *I. cairica* each contain three VOZ members, whereas *I. nil, I. triloba, I. trifida*, and *I. batatas* contain two each, indicating a contraction in VOZ gene copy number during the evolutionary diversification of the genus (**Figure 2A**; **Supplementary Table 1**). Furthermore, a comprehensive phylogenetic analysis incorporating 14 *Ipomoea* VOZ proteins together with two *A. thaliana* VOZ homologs demonstrated that IbVOZ1 clusters closely with ItbVOZ1, whereas IbVOZ2 shows a close evolutionary affinity to ItfVOZ2 (**Figure 2B**). Based on the resulting phylogenetic topology, VOZ proteins were categorized into three distinct clades: Group I (IaqVOZ1, IcaVOZ1, InilVOZ1, ItbVOZ2, ItfVOZ2, and IbVOZ2), Group II (IaqVOZ3 and IcaVOZ3), and Group III (IaqVOZ2, IcaVOZ2, InilVOZ2, ItfVOZ1, ItbVOZ1, and IbVOZ1) (**Figure 2C**).

### 3.3 Gene structure and conserved motif analysis of *VOZ*s in the six *Ipomoea s*pecies

**Figure 3:**
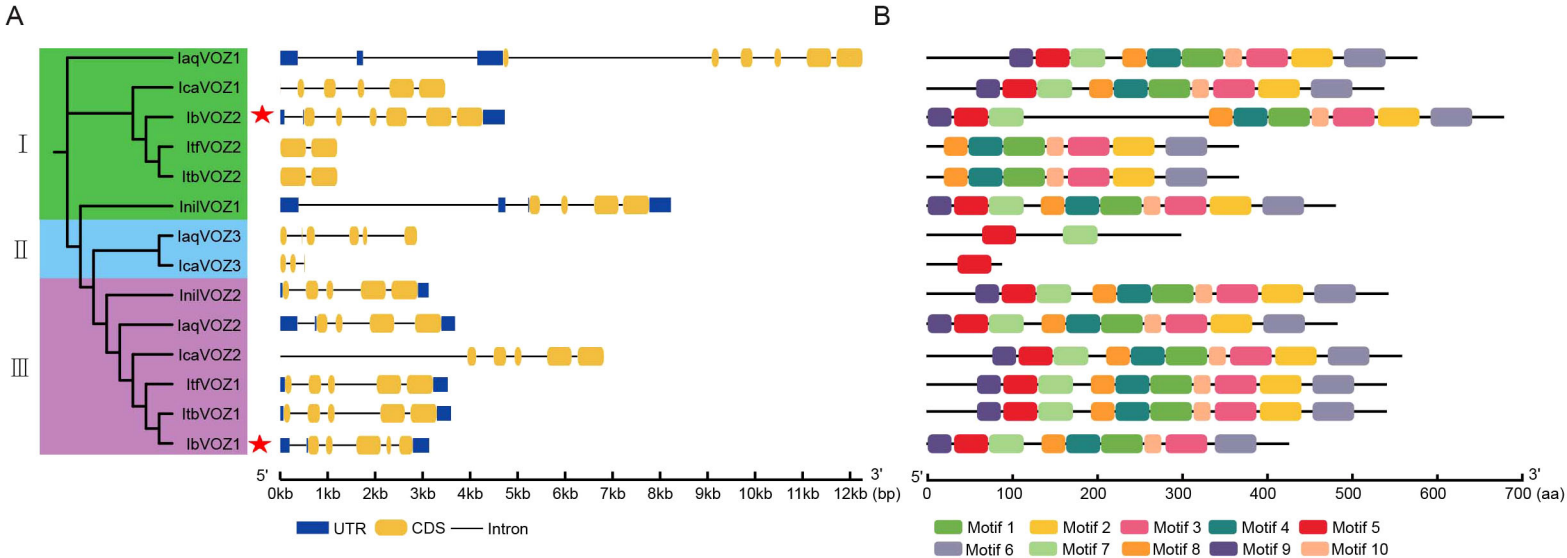
Gene structure and conserved motif analysis of *VOZs* in *I. aquatica*, *I. cairica*, *I. nil*, *I. triloba*, *I. trifida* and *I. batatas*. **(A)** The phylogenetic tree shows that VOZs were divided into three subgroups on the left. These genes were divided into three groups according to the evolutionary tree (Groups I, II and III filled with green, blue and purple, respectively). Gene structures of *VOZs* are shown on the right. Blue boxes, yellow boxes, and black lines represent the untranslated regions (UTRs), coding sequences (CDS) and introns, respectively. The red stars represent *IbVOZs*. **(B)** The ten conserved motifs were shown in different colors.

Gene architecture analysis offers valuable insights into the evolutionary trajectories and functional diversification of gene families. The organization of untranslated regions (UTRs), CDS, and introns within VOZ genes was systematically examined across six *Ipomoea* species. Most VOZ genes harbor either five introns (*InilVOZ2*, *ItfVOZ1*, *ItbVOZ1*, and *IbVOZ1*) or six introns (*IaqVOZ1*, *IaqVOZ3*, *IcaVOZ*1, *IcaVOZ2*, and *IbVOZ2*). Additionally, *ItfVOZ2* and *ItbVOZ2* comprise two CDS segments, *IcaVOZ3* contains three CDSs, and *IaqVOZ2* and *InilVOZ1* comprise four CDS regions (**Figure 3A**). The conserved sequence motifs within the *VOZ* proteins were identified using the MEME approach. The analysis revealed that individual *VOZ* proteins possess between one and ten conserved motifs. Notably, IcaVOZ3 contains only Motif 5, whereas IaqVOZ3 contains Motifs 5 and 7. Both ItfVOZ2 and ItbVOZ2 exhibit seven motifs (Motifs 1, 2, 3, 4, 6, 8, and 10). IbVOZ1 only lacks Motif 2, other VOZ proteins retain the complete set of 10 motifs (**Figure 3B**). Collectively, these findings indicate that the evolutionary history of the *VOZ* gene family has been influenced by variations in gene structure and motif composition.

### 3.4 Collinearity analysis of *VOZs* in the six *Ipomoea* genomes

**Figure 4:**
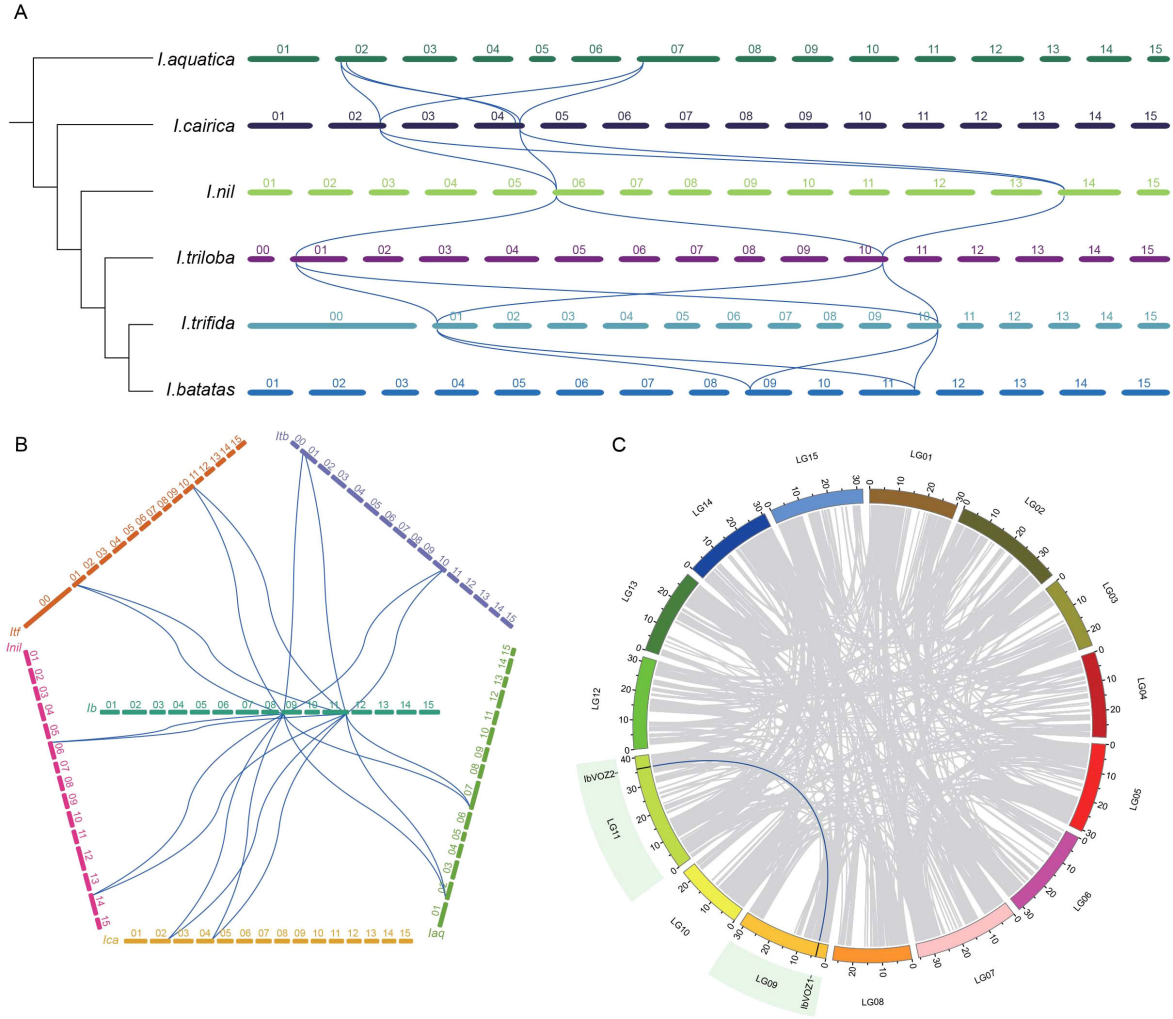
Collinearity analysis of VOZs in (I) aquatica, (I) cairica, (I) nil, (I) triloba, (I) trifida and (I) batatas. **(A, B)** Syntenic analysis of VOZs among the six Ipomoea species. Chromosomes of (I) aquatica, (I) cairica, (I) nil, (I) triloba, (I) trifida and (I) batatas are shown in different colors. Blue curves indicate the syntenic relationships of VOZs of the six Ipomoea species. **(C)** Chromosomal localization and distribution of VOZs in (I) batatas. The relative chromosomal localization of each VOZ gene is marked on the short black lines. Blue curves indicate the collinearity relationship between IbVOZ1 and IbVOZ2.

Comparative collinearity analysis across several genomes provides insight into gene family evolution and species divergence (Tang et al., 2008). To elucidate the evolutionary dynamics of *VOZ* genes within *Ipomoea*, collinearity analyses were conducted based on established phylogenetic relationships. Three conserved collinear blocks were identified between *I*. *aquatica* and *I. cairica*: *IaqVOZ1*–*IcaVOZ1*–*IcaVOZ2, IaqVOZ2–IcaVOZ1–IcaVOZ2*, and *IaqVOZ3*–*IcaVOZ3*. In addition, one intragenomic collinear gene pair (*IaqVOZ1*–*IaqVOZ2*) was detected in *I. aquatica*. These patterns indicate that the ancestral *Ipomoea* lineage may have had two VOZ genes, one of which experienced a duplication event in *I. aquatica* and was subsequently transmitted to *I. cairica*. During later evolutionary processes, IcaVOZ3 was not retained in *I. nil*, demonstrating the loss of genes in this lineage. In contrast, *IcaVOZ1* and *IcaVOZ2* were preserved and inherited by *I. nil, I. triloba, I. trifida*, and *I. batatas* (**Figure 4A**). To further assess the conservation of *VOZs* in sweet potato, collinearity analyses were performed between *I. batatas* and other *Ipomoea* species. Extensive collinear relationships between *IbVOZs* and their counterparts in related species indicate that the VOZ gene family in sweet potato has a high degree of evolutionary conservation (**Figure 4B**). A collinear relationship was detected between *IbVOZ1* and *IbVOZ2*, indicating that both genes arose from a shared ancestral locus. *IbVOZ1* and *IbVOZ2* are located on LG09 and LG011, respectively (**Figure 4C**). Duplication mode analysis using the duplicate_gene_classifier module of MCScanX revealed that whole-genome duplication (WGD) or segmental duplication is the predominant mechanism driving VOZ gene expansion in *Ipomoea*, with dispersed duplication contributing to a lesser extent (**Supplementary Table 1**).

### 3.5 Promoter *cis*-element profiling of *VOZs* across six *Ipomoea* species

**Figure 5:**
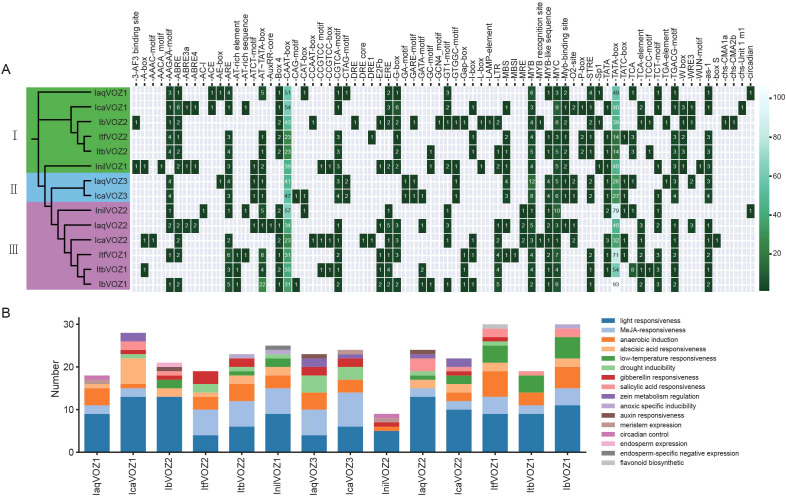
*Cis*-element analysis in the promoters of *VOZs* in *I. aquatica, I. cairica, I. nil, I. triloba, I. trifida* and *I. batatas*. **(A)** The intensity of green represents the number of *cis-*elements in the promoters of *VOZs.***(B)** The number of stress/responsive *cis*-elements of *VOZs’* promoters.

Promoter analysis provides insights into gene regulation and functional roles. Typically, *cis*-regulatory elements within promoters mediate responses to hormone signaling and stress stimuli (Hernandez-Garcia and Finer, 2014). To elucidate the regulatory mechanisms of VOZ genes, 2000-bp sequences upstream of the CDS were extracted for all *VOZs* in the six *Ipomoea* species, and *cis-*element prediction was performed. Several core promoter elements and TF binding sites were identified, with CAAT and TATA boxes being the most prevalent (**Figure 5A**). Functional annotation classified these *cis*-elements into three primary classes encompassing 16 distinct types. First, growth and development-related elements included motifs associated with zein metabolism, meristem expression, circadian regulation, endosperm expression, endosperm-specific negative regulation, and flavonoid biosynthesis. Second, environmental responsiveness elements encompassed motifs responsive to light, anaerobic induction, low temperature, drought, and anoxia. Third, hormone responsiveness elements comprised motifs responsive to Methyl jasmonate, abscisic acid, gibberellin, salicylic acid, and auxin (**Figure 5B**).

### 3.6 *VOZ* expression analysis in sweet potato

**Figure 6:**
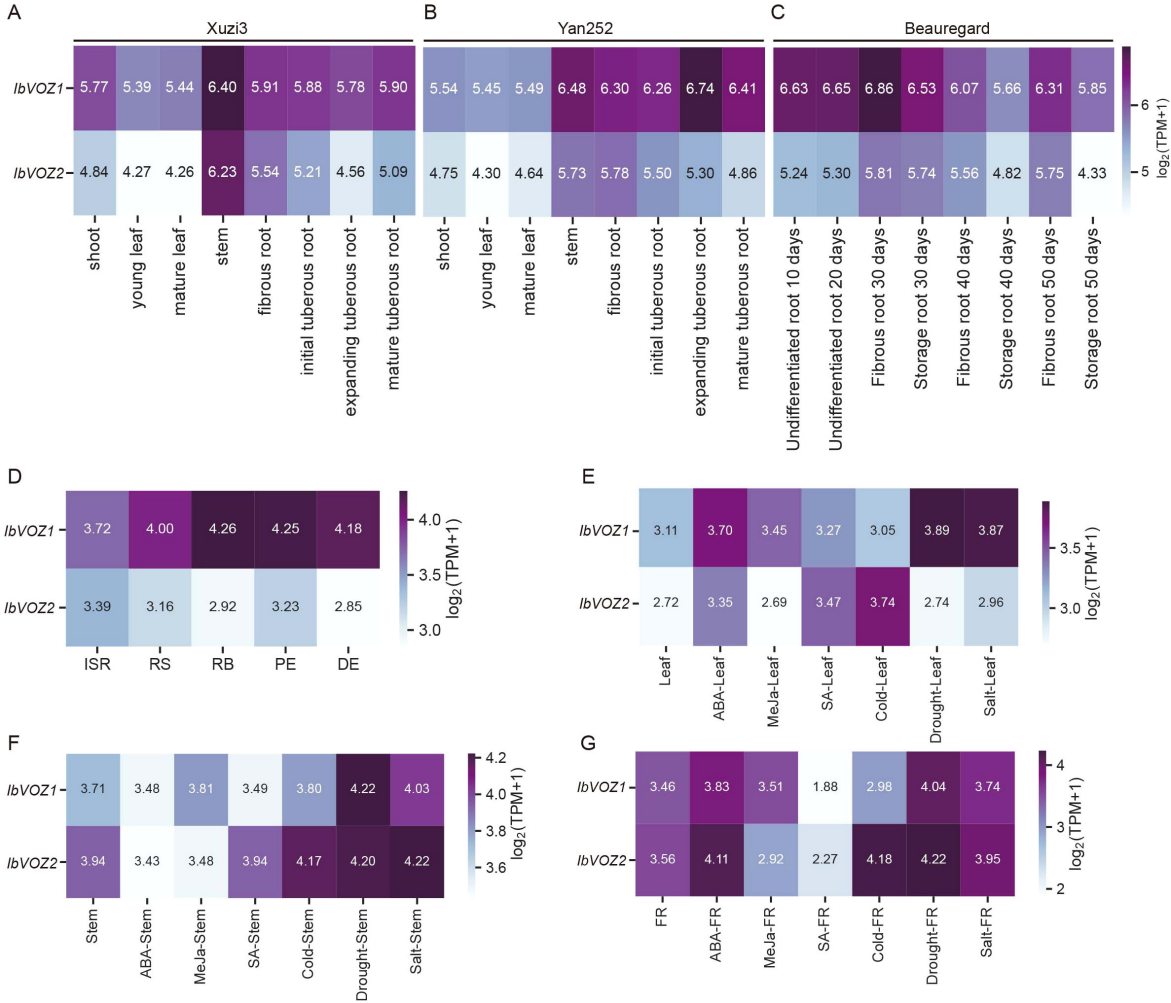
Gene expression patterns of *IbVOZs* in different tissues and under various treatments of sweet potato. **(A, B)** Gene expression patterns of *IbVOZs* in different tissues (shoot, young leaf, mature leaf, stem, fibrous root, initial tuberous root, expanding tuberous root and mature tuberous root) of ‘Xuzi3’ and ‘Yan252’. **(C)** Gene expression patterns of *IbVOZs* at different developmental stages of the fibrous roots and storage roots of ‘Beauregard’ (i.e., 10, 20, 30, 40 and 50 days). **(D)** Gene expression patterns of *IbVOZs* of different parts of the roots (ISR, initial storage roots; RS, root stalks; PE, proximal ends; RB, root bodies; DE, distal ends). **(E–G)** Gene expression patterns of *IbVOZs* in response to different phytohormones and stresses (i.e. ABA, MeJA, SA, cold, drought and salt) in leaves, stems and fibrous roots, respectively.

To explore the potential functional roles of *IbVOZs*, their tissue-specific expression patterns were examined in sweet potato, as spatial expression provides insights into gene function (Goring, 2012). RNA-Seq datasets from Ding et al. (2017) were analyzed across eight tissues in Xuzi3 and Yan252 cultivars: shoot, young leaf, mature leaf, stem, fibrous root, initial tuberous root, expanding tuberous root, and mature tuberous root. *IbVOZs* are expressed in multiple tissues, with the highest expression observed in Xuzi3 stems. In Yan252, *IbVOZ1* exhibited peak expression in stems, whereas *IbVOZ2* exhibited maximal expression in fibrous roots (**Figures 6A, B**). Given the importance of sweet potato as a root crop, IbVOZ expression during root development was further assessed using data from Coordinators (2018). *IbVOZs* were expressed at various root developmental stages, indicating roles in root formation and growth, with *IbVOZ1* consistently exhibiting higher expression than *IbVOZ2* (**Figure 6C**). Transcriptome data from Xu18 were analyzed to investigate expression during storage root development, including initiating storage roots (ISR), root stalks (RS), proximal ends (PE), and distal ends (DE) (Zhang et al., 2023). *IbVOZ1* exhibited maximal expression in the root body (RB), implying its involvement in the principal thickening phase, whereas *IbVOZ2* peaked in the ISR, indicating its role in early thickening (**Figures 6D**). In addition, *IbVOZ* expression was assessed in leaves, stems, and fibrous roots under abiotic stress conditions (cold, drought, salt) and hormone treatments (ABA, MeJA, SA). The results revealed differential induction of *IbVOZs* across tissues and treatments, highlighting their potential involvement in stress responses and hormone-mediated regulation (**Figures 6E–G**). Collectively, these findings revealed that *IbVOZs* play significant roles in root development and mediate responses to environmental stresses and hormonal signals (**Figure 6**).

### 3.7 Protein interaction network of IbVOZs in sweet potato

**Figure 7:**
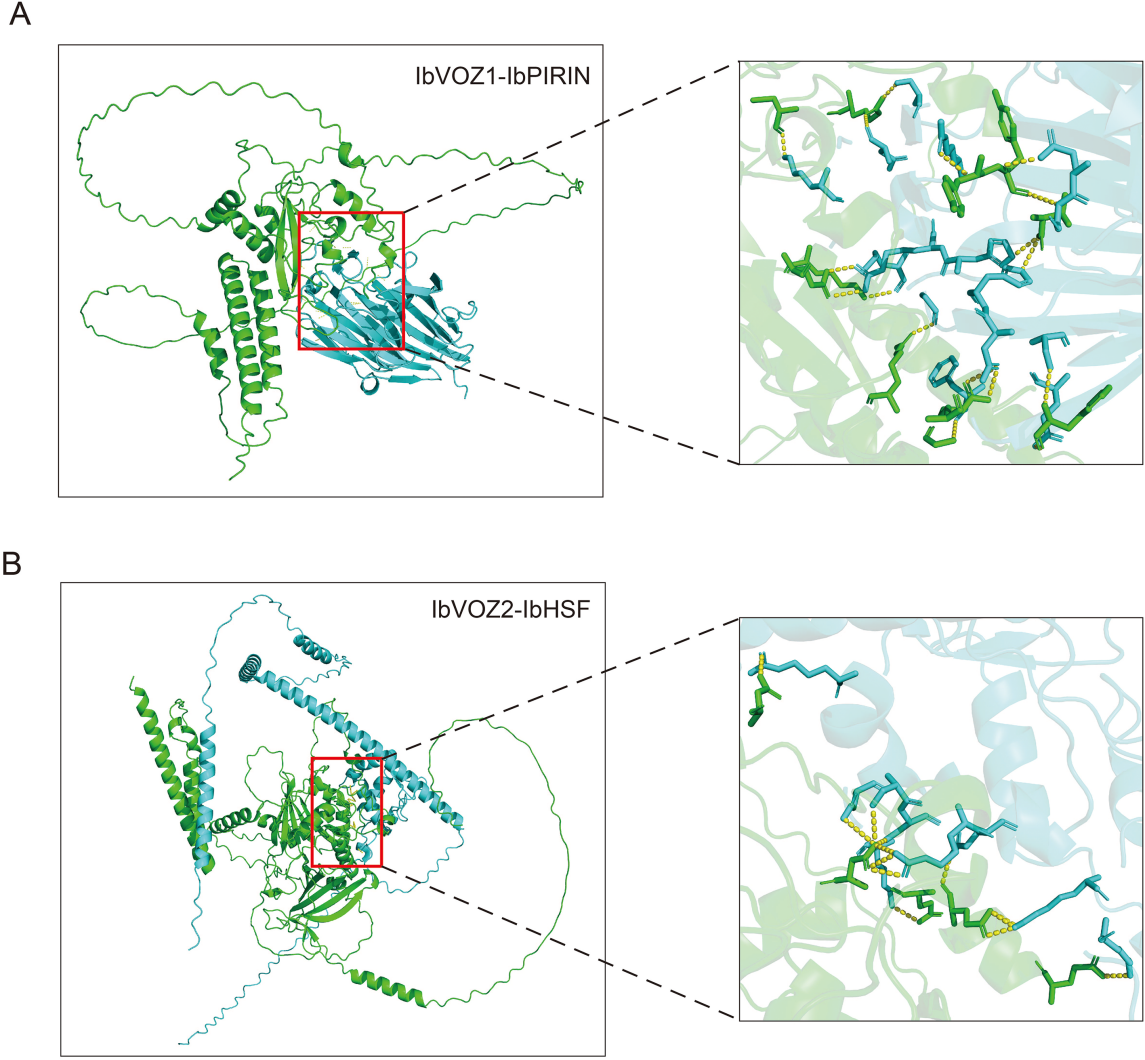
Protein-protein interaction networks of IbVOZs predicted by AlphaFold3. **(A)** Protein-protein interaction network between IbVOZ1 and IbPIRIN. IbVOZ1 is marked in green, IbPIRIN in cyan, and the red boxes indicate interaction sites. The right panel shows a magnified view of the interaction sites, with yellow dashed lines representing the interactions. **(B)** Protein-protein interaction network between IbVOZ2 and IbHSF. IbVOZ2 is marked in green, and IbHSF in cyan.

To elucidate the potential regulatory framework involving IbVOZs, genome-wide protein-protein interaction predictions were made for IbVOZ1 and IbVOZ2 (**Supplementary Figure 1**). The predicted interaction network indicated that IbVOZ1 is associated with IbPIRIN, IbWD40, and IbG-alpha, whereas IbVOZ2 is associated with IbHSF, IbPHY, and IbDSRM (**Supplementary Figure 1**). AlphaFold 3 was used to generate three-dimensional models of the interactions between IbVOZ1 and IbPIRIN and between IbVOZ2 and IbHSF. These structural models revealed the presence of multiple putative binding interfaces, providing structural support for the involvement of IbVOZs through protein-protein interactions in diverse biological processes (**Figure 7**).

### 3.8 IbVOZ1 encodes a nuclear and cell membrane-localized protein

*IbVOZ1* and *IbVOZ2* display intraspecific collinearity, indicating that they originated from a common ancestral gene (**Figure 4**). Comparative expression analyses showed that *IbVOZ1* is consistently expressed at higher levels than *IbVOZ2* across multiple tissues and developmental stages in different sweet potato cultivars (**Figures 6A–D**). Moreover, *IbVOZ1* exhibited a more pronounced transcriptional response to diverse hormonal and abiotic stress treatments, particularly salt and drought stress (**Figures 6E–G**). Based on these observations, *IbVOZ1* was selected for further functional investigation.

**Figure 8:**
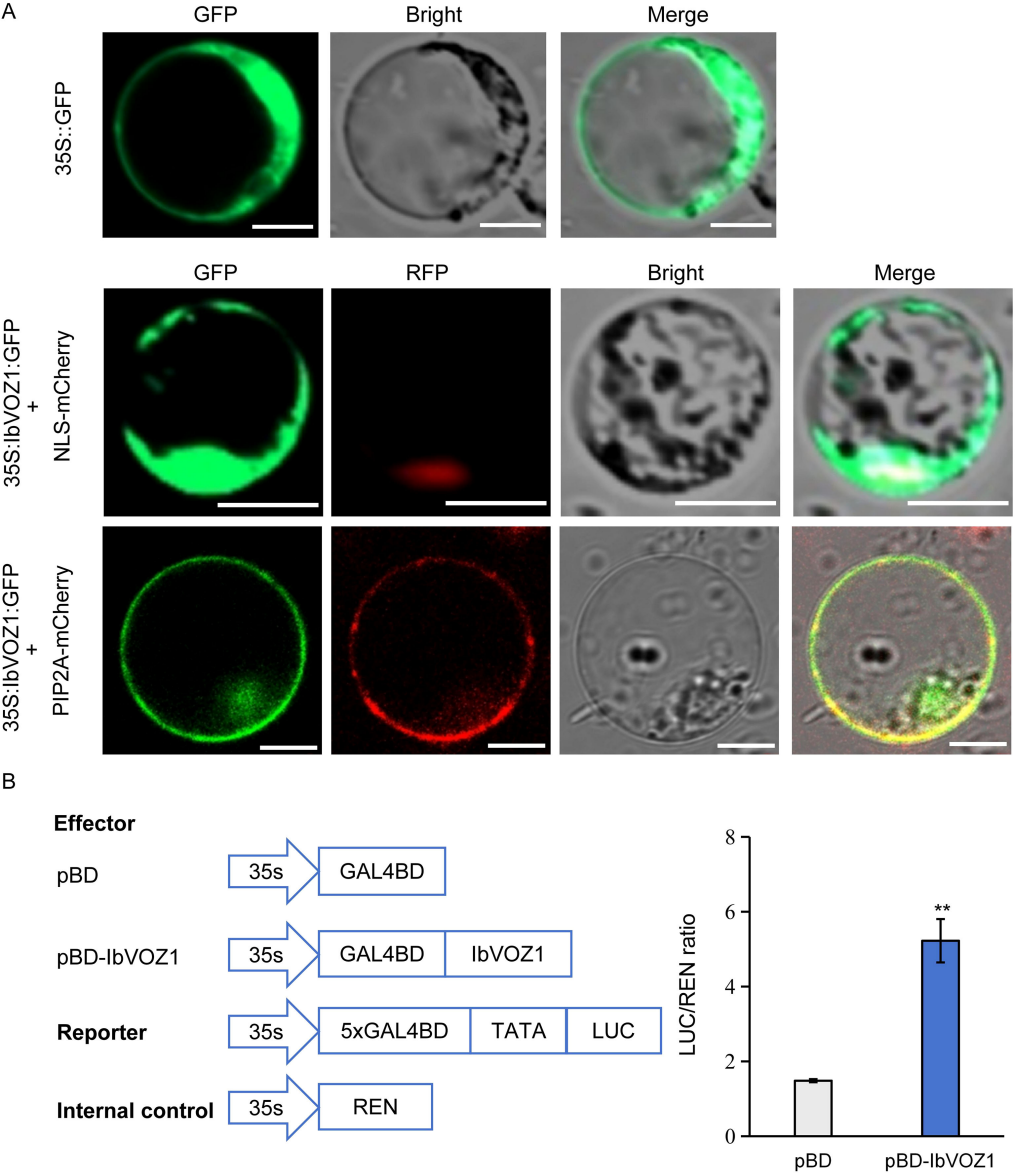
Subcellular localization and transcriptional activity of IbVOZ1. **(A)** Subcellular localization of IbVOZ1 in protoplasts. Green fluorescence indicates the IbVOZ1-GFP fusion protein, and red fluorescence indicates the markers NLS-mCherry (nucleus) and PIP2A-mCherry (membrane). Bar = 10 μm. **(B)** Transactivation assay of IbVOZ1 in protoplasts. The GAL4 BD empty vector was used as a negative control. The expression level of REN was used as an internal control. Error bars indicate SD (n = 3). **indicates a significant difference from that of pBD at *P *< 0.01, by Student’s t-test.

To determine the subcellular localization of IbVOZ1, transient co-expression assays were performed in protoplasts using an IbVOZ1-GFP fusion together with nuclear and plasma membrane marker proteins. Fluorescence microscopy analysis revealed the presence of IbVOZ1 in the nucleus and plasma membrane (**Figure 8A**).

### 3.9 IbVOZ1 as a transcriptional activator

The transcriptional activation potential of IbVOZ1 in protoplasts was evaluated using a dual-luciferase reporter assay. Effector and reporter constructs were co-expressed, and luciferase activity was measured after 16 h of incubation. Co-expression of IbVOZ1 resulted in a significant increase in reporter activity compared with control, indicating that IbVOZ1 possesses transcriptional activation activity (**Figure 8B**).

### 3.10 IbVOZ1 can be induced by 20% PEG6000 and 100 μM ABA

**Figure 9:**
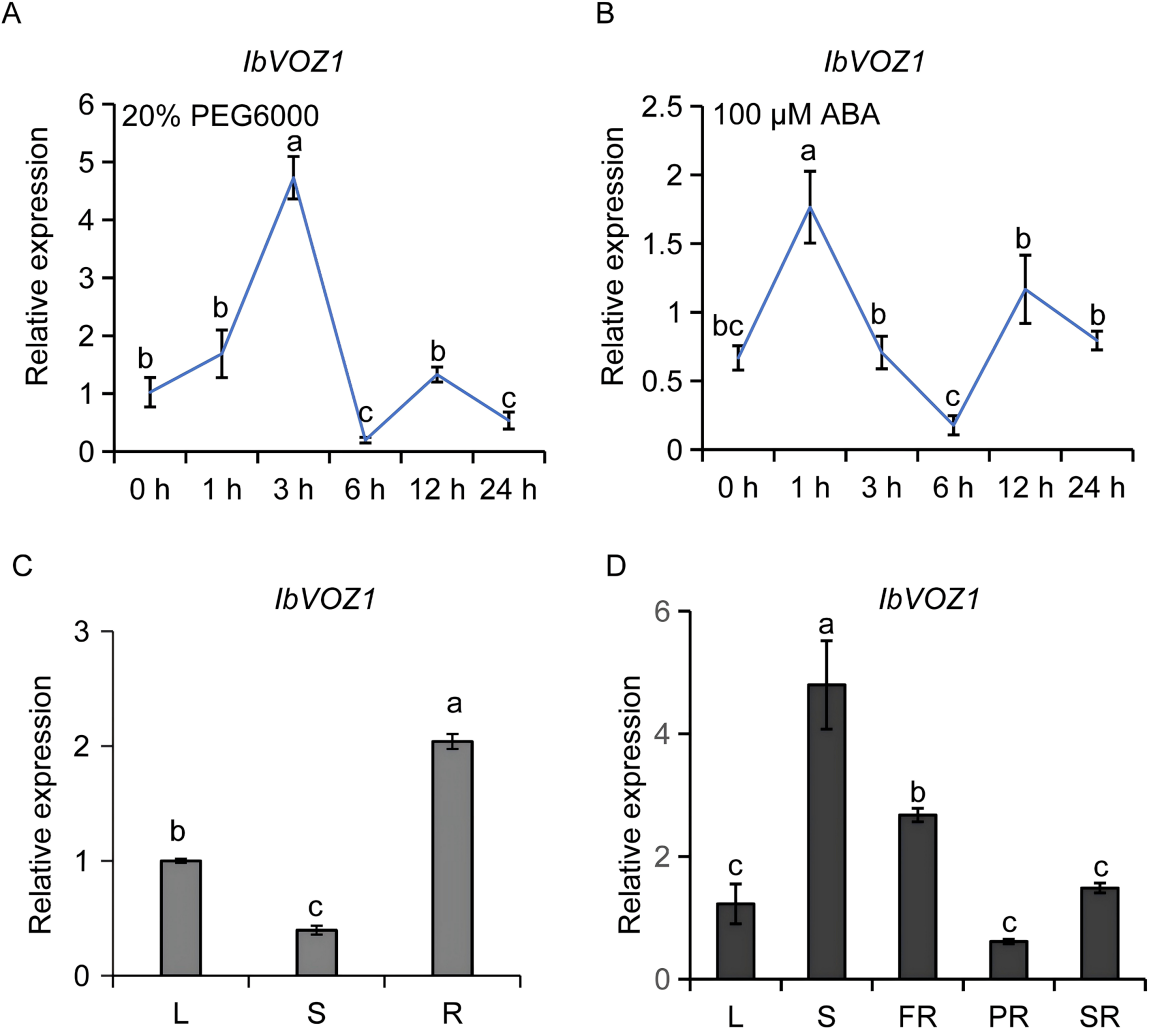
Expression analysis of *IbVOZ1* in sweet potato. **(A, B)** Expression of IbVOZ1 in 4-week-old *in vitro*-grown Xushu55–2 plants after different time points (h) upon exposure to 20%PEG6000 and 100 μM ABA, respectively. The sweet potato ACTIN gene was used as a reference. The expression level at 0 h in each treatment was considered as “1”. **(C)** Expression of *IbVOZ1* in the leaves (L), stems (S) and roots (R) of 4-week-old *in vitro*-grown Xushu 55–2 plants. **(D)** Expression of *IbVOZ1* in the leaves (L), stems (S), fibrous roots (FR), pencil roots (PR) and storage roots (SR) of 3-month-old field-grown Xushu55–2 plants. Data are shown as mean ± SD (n = 3). Different lowercase letters indicate significant differences at *P* < 0.05 based on one-way ANOVA followed by *post-hoc* Tukey’ s test.

RT-qPCR analysis demonstrated that 20% PEG6000 markedly induced *IbVOZ1* expression, reaching a maximum induction of 4.73-fold at 3 h after treatment. Similarly, exposure to 100 µM ABA significantly enhanced *IbVOZ1* transcript levels, showing a 1.77-fold increase at 1 h (**Figures 9A, B**). Tissue-specific expression profiling revealed that *IbVOZ1* exhibited the highest expression in the roots of 4-week-old *in vitro*-cultured Xushu 55–2 plants and in the stems of 3-month-old field-grown Xushu 55–2 plants (**Figures 9C, D**).

## 4 Discussion

Sweet potato is an economically important, high-yielding, nutritionally rich, and multifunctional root crop belonging to the genus *Ipomoea* (Liu, 2017; Kwak, 2019; Munoz-Rodriguez et al., 2019; Zhang et al., 2022). The functional characterization of sweet potato genes remains challenging because of its complex genome architecture. Nevertheless, recent advances in genome sequencing and assembly of *Ipomoea* species have provided valuable resources for examining the evolutionary dynamics and functional roles of genes in sweet potato (Hirakawa et al., 2015; Yang et al., 2017; Wu et al., 2018; Yan et al., 2022). Elucidating the evolutionary history of genes contributes to a better understanding of their biological functions. TFs serve as central regulators of plant growth, development, and environmental adaptation, and their identification and function are essential for understanding plant regulatory networks (Jack, 2001; Zhu et al., 2021). *VOZs* represent a plant-specific TF family that plays crucial roles in developmental regulation and stress responses. Although VOZ proteins were initially classified as a subfamily of NAC TFs, subsequent studies redefined them as an independent TF family (Jensen et al., 2010; Tian et al., 2020). The *VOZ* gene family has been characterized in several plant species, including six *GmVOZs* and four *CqVOZs* in soybean and quinoa, respectively. The evolutionary diversification of this gene family was elucidated by a comprehensive analysis of 107 *VOZ* genes across 46 plant genomes using integrated approaches (Gao et al., 2018; Li et al., 2020; Shi et al., 2022). However, the *VOZ* gene family has not yet been systematically investigated in sweet potato.

In this study, 14 *VOZ* genes were identified across six *Ipomoea* species. *VOZs* were distributed as three members each in *IaqVOZs*, *IcaVOZs*, and *InilVOZs* and two members each in *ItbVOZs*, *ItfVOZs*, and *IbVOZs*. These differences in gene copy number imply lineage-specific retention or loss within the *VOZ* gene family. In addition, variations in genome assembly quality, annotation approximations, and the intricate polyploidy history of *Ipomoea* species may underlie the observed discrepancies in *VOZ* gene counts. IbVOZ1 was closely associated with ItbVOZ1, whereas IbVOZ2 was closely associated with ItfVOZ2 (**Figure 2B**). VOZ3 members were the most phylogenetically divergent relative to the VOZ1/2 clades (**Figure 2C**), indicating that *VOZs* have experienced selective pressures over evolutionary time (**Figure 2**). Subcellular localization predictions indicate that most VOZ proteins are predominantly nuclear, with the exception of InilVOZ2, ItbVOZ1, and ItfVOZ1, supporting their proposed roles as transcription factors involved in nuclear gene regulation (**Supplementary Table 1**).

Structural analyses further illuminated the phylogenetic relationships and functional diversification of VOZs (Li et al., 2016). VOZ3 genes lacked UTRs and contained either three exons (IcaVOZ3) or six exons (IaqVOZ3), indicating that coding regions were more conserved than UTR sequences (**Figure 3A**). In contrast, ItbVOZ2 and ItfVOZ2 contained only two exons each, whereas IbVOZ2 contained six exons, indicating divergent structural evolution across species. Motif composition provides additional insights into gene regulation and function (D’Haeseleer, 2006). VOZ3 proteins contained only one or two motifs, VOZ1 proteins had nine or ten motifs, and VOZ2 proteins contained seven to ten motifs. VOZ3 consistently exhibited fewer motifs than VOZ1/2 across all six *Ipomoea* species (**Figure 3B**), and VOZ3 appears to have been lost in certain lineages. These findings indicate that a higher motif number may enhance functional versatility and adaptability, thereby favoring their retention through evolution. IbVOZ1 lacks motif2 compared with ItbVOZ1, and ItfVOZ2 lacks motifs 5, 7, and 9 although IbVOZ2 possesses all 10 motifs. Collectively, these findings demonstrate that VOZ genes have undergone structural modifications to optimize their functional roles and environmental adaptability throughout their evolutionary history.

Collinearity analysis has emerged as a pivotal approach for elucidating the evolutionary dynamics within the genus *Ipomoea*. MYB genes identified across seven *Ipomoea* species displayed high levels of synteny, implying derivation from a common ancestral gene (Si et al., 2023b). Conversely, nucleotide-binding site (NBS)-encoding genes in four *Ipomoea* species exhibited minimal collinearity, reflecting extensive genomic rearrangements following divergence from a shared ancestor (Si et al., 2022). Analysis indicates that *VOZ* genes in *Ipomoea aquatica* originated from two ancestral *VOZ* genes: *IaqVOZ1* and *IaqVOZ2* were derived from one ancestral gene, whereas *IaqVOZ3* arose from a distinct ancestral copy. *IaqVOZ3* was inherited by *I. cairica* during subsequent evolution; however, *IcaVOZ3* was not transmitted to *I. nil* and was subsequently lost. In contrast, *IcaVOZ1* and *IcaVOZ2* were conserved across other *Ipomoea* species. Collinearity mapping revealed that *IbVOZ1* and *IbVOZ2* in sweet potato displayed synteny, reflecting the strong conservation of *VOZ* genes (**Figure 4**).

During plant evolution and adaptation, transcriptional regulation is central to controlling key physiological processes. The diversity and abundance of *cis-*elements and the TFs that bind to them determine this regulation (Arnone and Davidson, 1997; Abnizova et al., 2007). Promoter *cis-*elements, typically spanning 5–15 bp, function as primary regulatory sequences for gene expression (Zhang et al., 2016; Yocca and Edger, 2022). Analysis of *VOZ* gene promoters revealed the presence of *cis*-acting elements linked to plant growth, development, and stress responses, including motifs responsive to auxin, gibberellin, jasmonic acid (JA), and ABA (**Figure 5**). ABA-responsive elements (ABREs), which are recognized by ABRE-binding factors (ABFs), are critical for modulating gene expression under environmental stress (Hobo et al., 1999). Most VOZ promoters—including those of *IaqVOZ1*, *IaqVOZ2*, *IcaVOZ1*, *IcaVOZ2*, *InilVOZ1*, *ItbVOZ2*, *ItfVOZ2*, *ItfVOZ1*, *IbVOZ1*, and *IbVOZ2*, contained ABREs (**Figure 5**), consistent with the upregulation of *IbVOZ* transcripts in roots and leaves following ABA treatment (**Figure 6**). These findings indicate that *VOZ* genes participate in ABA-mediated responses to abiotic stress (**Figures 5**, **6**) and are broadly regulated by multiple phytohormones essential for growth, development, and stress adaptation.

Potential interaction partners of IbVOZ proteins were predicted to elucidate their regulatory networks. IbVOZ1 was found to interact with IbPIRIN (**Figure 7**; **Supplementary Figure 1**), a protein previously shown to mediate stress responses in *Arabidopsis* (Orozco-Nunnelly et al., 2014; Brunetti et al., 2022). IbVOZ1 interacted with IbWD40 (**Supplementary Figure 1**). In rice, OsWD40–193 forms a complex with OseEF1A1 to suppress *Hirschmanniella mucronata* infection (Shan et al., 2023) and *IbWD40* overexpression enhances anthocyanin accumulation in transgenic *Arabidopsis* (Dong et al., 2014). IbVOZ2 interacts with IbHSF and IbPHY (**Figure 7**). Heat shock factors (HSFs) are well-established regulators of heat stress responses (Jiang et al., 2021), and overexpression of *TaHsfA2e-5D* improved thermotolerance in *Arabidopsis* (Bi et al., 2022). Phytochromes (PHYs) act as red/far-red light photoreceptors essential for light perception and developmental signaling (Smith, 2000; Hennig et al., 2002). These interactions demonstrated that IbVOZs may modulate diverse proteins to regulate essential physiological processes in sweet potato through dynamic protein-protein interactions. Furthermore, IbVOZ1 was experimentally confirmed to function as a transcriptional activator, with its expression strongly induced by PEG and ABA treatments (**Figures 8**, **9**). This finding confirms that IbVOZ1 functions as a TF with activation potential and establishes a conceptual basis for future functional investigations.

## 5 Conclusions

This study identified and characterized 14 *VOZ* genes in six *Ipomoea* species. Comprehensive analyses, including protein characterization, evolutionary and phylogenetic assessment, gene structure evaluation, conserved motif identification, collinearity mapping, and *cis*-element analysis in promoter regions, were performed. These investigations indicated that the VOZ genes clustered into three unique groups, with VOZ3 homologs lost during evolutionary history, and that *IbVOZs* originated from a common ancestral gene. Tissue-specific expression profiling, hormone- and stress-induced expression analyses, and protein-protein interaction networks were used to further elucidate the functional roles of IbVOZs in sweet potato. The functional characterization of *IbVOZs* demonstrated that they act as a transcriptional activator localized to both the nucleus and plasma membrane, with their expression induced by osmotic stress and ABA treatment. Collectively, these findings indicate that *IbVOZ* genes play critical roles in regulating growth, development, and stress responses in sweet potato. Identifying stress-responsive and hormone-regulated VOZ genes provides valuable candidate targets for genetic improvement. *IbVOZ1* represents a promising gene for enhancing drought tolerance, and its regulatory mechanisms may inform future breeding strategies. Further studies should investigate the downstream targets of *IbVOZ1* and other *VOZ* genes to construct a detailed drought tolerance regulatory network, paving the way for precision breeding and biotechnological applications in sweet potato.

## Data Availability

The original contributions presented in the study are included in the article/**Supplementary Material**. Further inquiries can be directed to the corresponding authors.
